# Australian private healthcare staff perspectives on patient reported experience measures (PREMs): a qualitative interview study

**DOI:** 10.1186/s41687-024-00809-6

**Published:** 2024-11-06

**Authors:** Krista Verlis, Kirsten McCaffery, Tessa Copp, Rachael Dodd, Brooke Nickel, Rebekah Laidsaar-Powell

**Affiliations:** 1 Sydney Health Literacy Lab, Sydney School of Public Health, Faculty of Medicine and Health, The University of Sydney, Sydney, NSW Australia; 2 Faculty of Medicine and Health, The Daffodil Centre, A Joint Venture Between The University of Sydney and Cancer Council NSW, Sydney, Australia; 3 Psycho-Oncology Cooperative Research Group (PoCoG), School of Psychology, Faculty of Science, The University of Sydney, Sydney, NSW 2006 Australia

**Keywords:** Patient reported experience measures, Decision-making, Hospital admission, Hospital report cards, Private health insurance, Qualitative

## Abstract

**Objective:**

Patient reported experience measures (PREMs) are common tools utilised in hospitals to support quality improvements, allow consumers to provide feedback on care experiences and can be used to support consumers’ hospital selections. This study aimed to understand the views and opinions of private hospital staff on PREM use and the utility of PREMs as a consumer decision-making tool.

**Method:**

Qualitative, semi-structured interview study conducted via telephone between March-June 2023. Participants (*n* = 10) were recruited from major private healthcare providers in Australia with half representing hospital-based staff and the other half corporate head office staff who work in patient experience and quality. Interviews were audio-recorded, transcribed, and analysed thematically.

**Results:**

PREM benefits included an understanding of patient experience that improved provision of patient centred care with feedback acting as catalyst for change, to corporate-level strategic initiatives that address specific issues. Drawbacks of PREM reporting included concerns around skewed results by biased respondents, and completion based on hard to alter items (e.g., infrastructure) or on matters outside of hospital control (e.g., insurance). Staff had mixed reactions to consumers using PREMs results when selecting a hospital, some advocated for transparency while others feared consumers would misinterpret the data.

**Conclusions:**

Improved real-time reporting of PREMs, learning from other industries about recording customer experience, and mandatory reporting by private hospitals could further the benefits of PREM measurement in private healthcare. Recognised was the need for PREMs to be displayed in a readily understood way so those with limited health literacy can correctly interpret.

**Supplementary Information:**

The online version contains supplementary material available at 10.1186/s41687-024-00809-6.

## Background

Patient Reported Experience Measures (PREMs) are increasingly utilised tools for improving quality of care provided in healthcare facilities [1, 2]. PREMs are defined as ‘*a measure of the views and observations on different aspects of the health care services that a person has received*’ [3, 4] and encompass aspects of the physical environment of where the services were provided as well as patient-clinician interactions [5]. When designed and administered appropriately, PREMs can provide indicators and opportunities to improve healthcare quality [6, 7] and provide information that can aide improved healthcare delivery [8].

More consistent and mandated reporting occurs by public hospitals in Australia, often via the Australian Medical Association hospital report cards and public performance reporting (PPR) on the Australian Institute of Health and Welfare My Hospitals website [9, 10]. Some larger private healthcare providers, individual hospitals and private health insurers do publish their own patient experience data on websites to inform consumers (e.g., Medibank Hospital Experience Scores website^1^) but these are inconsistent in what is presented. For instance, the PPR reported by private hospitals on the MyHospital^2^ website is voluntary and does not include PREM data [10] and those displayed on individual hospital websites can vary in the way review data is presented (e.g., with percentages and a metric [11] or posting positive written reviews [12]. This variability in disclosure and lack of consistency in, if and how, patient satisfaction results are displayed limits the utility of this information for consumers to make comparison or in making meaningful decisions. Mandated public hospital performance reporting in Australia often includes wait times for emergency departments and elective surgery and infection rates. This reporting aims to improve hospital safety through openness and transparency [5] and by assisting consumers in identifying high- and low-quality providers [10]. Most performance metrics, at least in Australia, do not currently report on PREMs data [10].

A global review indicated that hospital performance information released to the public showed a slight increase in improved patient outcomes but made little to no difference to healthcare choices (by both consumer and provider) or to provider performance [13]. Other studies contradicted this latter finding however, showing publicly available information stimulated quality improvement activities [14, 15]. The need for PREMs (and Patient Reported Outcome Measures (PROMs)) data to inform health systems is recognised as making healthcare more people-centred and facilitating provision of value-based healthcare [16, 17].

There is limited knowledge on the perspectives of Australian private healthcare organisations towards the use of PREMs as a consumer decision making tool and how collected PREMs could be improved. This study explored with hospital staff what patient experience data is currently collected, how private hospital organisations utilise and view PREM data and how PREM data could be improved to potentially be used by consumers.

## Methods

### Study design

This qualitative study used semi-structured telephone interviews to capture the views of Australian private healthcare organisation staff. This project received ethics approval from The University of Sydney Human Research Ethics Committee (2022/686). All participants provided written informed consent prior to participating.

### Participants

Staff members from major private healthcare groups which own and operate private hospitals in Australia were emailed an invitation to participate in the study with a request for staff to also forward the invitation to any relevant staff members. Participants from two key roles were recruited: (i) hospital-based quality improvement staff, and (ii) corporate / head office staff who manage a patient experience portfolio. Interested participants were emailed the Participant Information Statement and consent form and booked into an interview. Participants received a $50 retail gift voucher as compensation for their time. Participants were recruited until thematic data saturation was achieved, as indicated by no further original themes being raised in the data [18, 19].

### Data collection and analysis

Interview schedules were developed and iteratively refined among the authorship group comprising experts in health services, qualitative research, and consumer decision-making. Semi-structured telephone interviews were then conducted by a qualitative researcher (KV and RL-P), audio-recorded and transcribed verbatim (see Appendix A for full Interview Schedule). Demographic, professional, and health organisation information was collected. Interviews lasted an average of 32-minutes (range 19–46 min) and were conducted between March and June 2023. Open ended questions explored staff members experiences and thoughts on PREMs, the kind of PREMs utilised and how they were used. Transcripts were thematically analysed in NVivo 12 Software [20] using a framework analysis that identified recurring themes and data patterns [21] according to a five-stage process (identify initial themes; construct an index; label the data; sort the data by theme; and synthesise and interpret the data) [21]. Multiple authors (KV, BN, RL-P) were involved in the development of the coding framework to ensure consistency and methodological rigour with any disagreements resolved prior to implementation.

## Results

A total of 10 private hospital and health service staff participated in interviews (Table 1).

**Table 1:** Summary of participant characteristics (*n* = 10)

Characteristic	Total No. (%)
**Role**	
Corporate head office patient experience and clinical governance	5 (50%)
Quality and safety officer at private hospital	5 (50%)
**Size of private hospital**	
100–199 beds	3 (30%)
200–299 beds	2 (20%)
300–399 beds	1 (10%)
400 + beds	4 (40%)
**Hospital settings present**	
Emergency	7 (70%)
Maternity	8 (80%)
Intensive Care Unit	10 (100%)
**Time working as a healthcare professional** range (mean ± SD) in years	13–41 (27.8 ± 10.0)
**Time working in patient experience & quality management** range (mean ± SD) in years	2–22 (11.3 ± 7.3)
**Interview length** range (mean ± SD) in minutes	19–46 (32.0 ± 8.6)

Thematic analysis of interview data identified four overarching themes: (1) Usage of PREMs data within healthcare services; (2) Attitudes towards the collection, analysis, and reporting of PREM data; (3) PREM as a consumer decision-making tool, and (4) The future of PREMs.

### Theme 1: usage of PREM data within private healthcare services

#### Type and timing of internal PREM data

All participants utilised PREMs information in multiple ways to improve patient experiences and reported their private health organisation collected PREM data through internal processes. Many reported that their organisation used the Net Promoter Score (NPS) which is a single, standard item measured on a scale from 1 to 10 on how likely you are to recommend [healthcare service] to a friend or colleague? This measure is used by many hospitals in Australia, for the evaluation of patient experience [22] with optional open text comments.

Of the organisations that utilise the NPS, most staff discussed that this was sent to every patient after discharge, and they received “*almost real-time*” feedback. The reported benefit of the NPS was that it allowed faster identification and response to emerging issues; however, a negative was that results are “*not hugely detailed*”.

Some participants also discussed the value of free-text feedback comments collected through the NPS which could be analysed to identify specific areas.

We also use the free text comments that are provided to share that information with the teams, as well, so that we can identify very specific areas, if need be.

Additionally, most participants stated their organisation collected more comprehensive assessments using validated PREMs instruments such as the Australian Hospital Patient Experience Question Set (AHPEQS) or the Hospital Consumer Assessment of Healthcare Providers and Systems (HCAHPS) measure. The frequency of collection, analysis, and reporting internally, and the proportion of patients surveyed, varied widely according to the survey instrument and organisation. The level of sophistication in reporting and insights varied widely, with some staff describing basic as-needs styles of PREM data analysis, whereas others had custom dashboards permitting deeper insights into PREM data. Most participants noted the strengths and drawbacks of each measurement type and highlighted that their organisation used a combination approach to gain both rapid (NPS) and deep (PREM) insight into their healthcare service.

Of PREM data received from *other* organisations, Medibank (private insurer) was the most frequently identified external source. Some participants also received PREM data from the Department of Veterans Affairs or from the NSW Health Bureau of Health Information that reports on Patient Experience Surveys among specific clinical groups (e.g., oncology).

#### Who sees and uses PREM data in private hospitals

Participants described a wide variety of corporate and hospital-based staff members who viewed both internally collected and external PREMs information. This included hospital-based leadership and committees, such as the Hospital General Manager.

Every time we get a source of data, we share it with key governance committees We also [share PREMs] with all our leaders, not all of those are clinical but we’ll raise with them too. Because patient experience is not just about the clinicians. Everyone who sees the patient can impact on that.

At the hospital-site level, participants described how PREMs are analysed and actioned. Engagement was particularly prevalent amongst staff who have access to real-time data. Some noted that the NPS data was viewed by quality and safety officers daily, with more comprehensive PREM data and reporting reviewed on a weekly/monthly basis. They noted that PREM information was shared with clinical leaders who then distributed to broader clinical teams.

We have a bed management meeting every morning … The [higher level manager] pulls up [online PREMs portal] where the AHPEQs is. Then, there is a monthly meeting … all the nurse unit managers report into that meeting, to their peers and to the [higher level management].

A couple of participants also noted that PREM information was reviewed by consumer advisory groups, who assisted in developing improvement strategies.

### Theme 2: the perceived benefits and challenges of PREM data

Participants stated deep commitment to high quality patient-centred care and the improvement of patient experiences. All held strongly positive attitudes towards PREMs collection/analysis/reporting and spoke of PREMs as a valuable tool to monitor and improve patient experiences. Though some challenges were noted. See Table 2 summary of perceived benefits and challenges.

**Table 2:** Perceived benefits and challenges of PREM collection and use

Factor	Supporting Quote
**Perceived benefits**	
**PREMs as the patient voice**: Provide an avenue for patient feedback and an understanding of the patient experience	*“If we’re providing person-centered care*, *and it’s*, *‘Nothing about me*, *without me’ then our major driver is our PREMs*.”
**Timely surveillance of care quality**: Frequent PREMs reporting provides timely insights into performance and emerging issues.	*“Two weeks after discharge is too late for us. We need the ability to be able to survey our patients and pulse check – at point-of-care – to be able to immediately respond to our consumers.”*
**Prioritise areas of improvement and strategic initiatives**: Can act as a catalyst for improvement projects, hospital/organisational change, targeting areas that patients value, insights for hospital/group wide initiatives, and benchmarking against peers.	*“Unless we know what’s important to patients and are measuring that*, *we might be easily offering the wrong services and the wrong pathways … PROMs and PREMs are very important from the value-based healthcare perspective.”*
**Effectiveness of introduced change(s)**: Useful in monitoring the effectiveness of organisational change or specific improvement projects.	*“We want to be the best at what we do and we need to know how it’s being perceived by and received by patients so that we can make sure we’re doing the right quality improvement projects.”*
**Staff recognition**: Celebrating staff members and teams who are providing excellent patient experiences; Though not every participant agreed with overtly celebrating of PREM scores.	*“I think it’s certainly good to celebrate that we’re doing well. But I just don’t know that we need to celebrate treating people well. The bar’s really low if we have to celebrate treating people well.”*
**Peer education and support**: Enable the sharing and leveraging of strategies from high performing teams across the healthcare organisation.	*“Making sure that our teams are aware of what the results are and identifying if it’s something that we’re doing well at*, *sharing that…getting them to share with the other areas to improve across the board.”*
**Perceived challenges**	
**Recruitment and Response Rates**: Possible issues resulting in biased data or inaccurate representation of patient experiences, such as being skewed to represent very satisfied/dissatisfied patients.	*“there’s a fair amount of survey fatigue…. Getting richness of data is problematic*, *because people can’t be bothered with it*, *unless they’ve got something that’s really important to say.”*
**Demographic representation**: Particular groups may not engage with surveys, therefore PREMs may not represent the whole patient cohort.	“*A lot of elderly patients are not going to like receiving SMS’*, *or emails*, *they want a paper version”*
**Subjective nature of experience**: Survey completion could be unreliable and subjective - with some patients not using the scoring system as intended.	*“There’s not a lot of sensitivity around the score that’s one to ten*, *and people are so used to these rating systems now that it becomes a little flippant.”*
**Viewpoint**: When family complete PREMs, not the patient, data reliability may be compromised.	*“And as long as it’s the patient filling [PREMs] in*, *and not a relative*, *because that also changes it as well.*
**Increased workload and capacity issues**: The cost of collecting, analysing, reporting, and acting on PREMs can be time consuming. Some private hospitals feel ill-equipped to manage all of the data.	*“Gold miners had to sift a lot of gravel before they found the specks of gold. It’s a little bit similar. You often have to go through a fair bit of gravel before you actually get to the real crux of the themes*, *the genuine problems*, *to make substantive change.”*
**Staff pushback**: Some hospital staff have negative perceptions of PREMs feedback, were resistant to change, or may not value the transparency of PREMs.	*“Any type of change management is tricky. Especially when you have had people who work within facilities for a long time and don’t particularly want to change”.*
**Limitation on reach**: Feedback on hard-to-change issues to be difficult to manage, such as hospital infrastructure.	*“There’s stuff we can’t control … we don’t have single rooms everywhere*, *or not every patient room has a toilet dedicated to that room…”*
**Unrelated items being considered**: Some participants experiences were unfairly attributed to the hospital, for instance because of misunderstanding their level of private healthcare coverage or having unanticipated out-of-pocket expenses.	*“Alot of people will complain and give a poor rating because their emergency department care wasn’t funded by their health insurance. Now*, *that that comes back on [PREM] measure as a problem for the hospital*, *but it’s not a problem for the hospital.”*
**Staff morale**: Poor scores or negative feedback can be difficult and upsetting for clinicians to receive	*“When you go and take a piece of feedback to someone on a ward*, *whether it’s a nurse or a doctor*, *they are a little bit defensive. But generally they will take it [feedback] on”*

### Theme 3: PREMs as a consumer decision-making tool

Participants expressed mixed perspectives on PREMs being used by consumers to inform hospital choice. Most held positive attitudes towards the idea of PREMs being available to help consumers choose a hospital, and valued transparency in healthcare. One participant stated *“[I] Love [PREMs for consumers]. I’m big on transparency with everything…. people make their choices based on that”.* However, some had reservations for when consumers had little choice in hospitals or had low health literacy. These views are summarised in Table 3.

**Table 3:** Perspectives on PREMs as a consumer decision-making tool

Factor	Supporting Quote
**Researching decision as the norm**: Researching other people’s experiences is what consumers now do across many industries, and healthcare is no different. Increased health consumer empowerment has resulted in more patients wanting information to make informed decisions.	*“We know that as consumers in other sectors*, *we all individually often look up websites to see what’s recommended and what the star ratings are*, *of a certain restaurant or whatever. It is important to consumers*, *and we do need to be able to give them good data that they can understand*, *and that’s fair and reasonable”.*
**Helpful when abundance of choice**: PREMs as a consumer decision-making tool was particularly useful when there is a lot of choice, such as living in a metropolitan area with multiple private hospitals nearby.	*“…if I was in a city with seven different hospitals*, *absolutely I would use it as one measure.”*
**Problematic if limited choice**: For regional and rural Australians, they may only have one nearby hospital so this information may have negative repercussions.	*“I think if [public availability of PREMs] is not managed well*, *it’s dangerous. Where you’ve got rural and regional Australians who have got one community hospital*, *and that may be their only choice around there*, *you don’t want to do something that potentially compromises the care of patients because they choose to have to travel another 100 km down the road*, *because we think the colour of amber [on PREMs website] on our local hospital isn’t as good as the colour of green down the road.”*
**Irrelevant when there is no choice**: PREMs information may not be relevant to some consumers as their doctor may only operate at one hospital or patients prefer to follow their doctor’s recommendation for the hospital.	*“A lot of patients choose the place that they’re familiar with*, *that they know has a good reputation*, *but in a lot of instances it’s what their doctor recommends.”*
**Need for health literacy**: While transparency and informed decision-making was supported, some were wary of issues with this information being made available to the public. Some consumers have lower health literacy and may misinterpret the information. Data needs to be displayed in a clear, easy-to-interpret way and that consumers understand the methodology behind the PREM.	*“There’s a lot of challenges with health literacy and understanding the differences between hospitals. There’s a lot of challenges with them understanding what the data means and have we really presented in a way that they would be able to compare apples with apples.”*
**Potential for reputational damage**: May be daunting for hospitals who have negative PREM results to publicly make it available for consumers, as it may impact the hospital’s reputation and business.	*“I guess it could be daunting if you’ve got negative results for your hospital because that’s not great for business*, *but I believe in transparency for patients*, *they’re entitled to know these things.”*
**Importance of clinical outcomes**: Less in favour of PREM reporting with more emphasis placed on patient outcomes, as these were perceived to be more important.	*“The patient-reported experience measures has to be countered a little bit by what some of the outcomes are for hospitals. So you might go*, *“Okay*, *well*, *they haven’t got great food*, *but gee*, *they’ve got a very low rate of stillborn babies at this hospital*, *or progression to Caesar*, *or needing an episiotomy.” I’d be looking at the clinical outcomes.”*

### Theme 4: the future of PREMs

A few participants reflected on how far PREMs had come over their careers, while also acknowledging room to improve. Planned improvements for the collection, analysis, and reporting of PREM data were reported across different organisations, particularly for those that did not have detailed analytics or year-round insights. Some thought more real-time PREMs information could facilitate more immediate responses. One participant planned to deliver standardised education to staff on how to effectively use PREMs data and insights.

Some participants discussed opportunities to look outside of healthcare to gain inspiration and ideas on measuring experience.

I think there’s probably an opportunity to look at what other organisations outside of healthcare do from an experience measure … I think there’s opportunities even within our organisation to look at … how we get patient experience feedback. How do we make it so that it’s not siloed?

Two participants advocated for National PREMs reform. Both suggested the Australian Commission of Safety and Quality in Healthcare (ACSQHC) mandate a standardised reporting and benchmarking system of PREMs across both public and private healthcare sectors. One participant believed that the PREMs obtained through this system would have more “*gravitas*” and benchmarking would be more widely accepted by clinical staff if it came from a Government initiative.

I think it’s the underpinning foundational data piece nationally we should be measuring in a consistent and good way. I wish the ACSQHC were making a national portal for people to submit their AHPEQS or something and that there was a national benchmark available from the Safety and Quality Commission. And it should be a national initiative, PREMs and PROMs done by the Commission.

## Discussion

This qualitative interview study of Australian private healthcare organisation staff provides insight and opinion on the perceived benefits and drawbacks of PREMs and the possible use of PREMs as a decision-making tool for consumers. PREM benefits included an understanding of patient experience that improved the provision of patient centred care with feedback acting as a catalyst for change, from corporate-level strategic initiatives to addressing specific issues at hospital sites. These are therefore similar to PROMs which are also used in quality improvement and improving patient care, but PROMs are thought to be more subjective. PROMs measure the patient’s view of their health status and allow for the patient perspective to measure the efficacy of any clinical intervention [1, 23]. Drawbacks of PREM reporting included concerns around skewed results by biased respondents or by surveys being completed by carers and not patients themselves. Completion based on hard to alter items (such as infrastructure) or on matters outside the control of the hospital (such as insurance coverage) were raised. Also raised were the resistance of some staff to change and issues around morale and response to negative criticisms. Improved real-time reporting of PREMs, learning from other industries about recording customer experience and the mandatory reporting of PREMs by private hospitals could further the benefits of PREM measurement in private healthcare.

The overall attitudes of private healthcare staff captured in this study (who represented quality improvement and corporate staff) were largely positive towards PREM collection and reporting (at least internally) with most having a positive view on public reporting. This is important as research from the USA, demonstrates senior leadership plays an important role in a healthcare organisations quality improvement culture [14]. However, there is concern by many hospital staff around patient health literacy in understanding and interpreting the data, as well as around the quality and age of the data made publicly available to reflect current practices [14]. An earlier Australian study reported half of respondents indicated an institutional cultural resistance to public reporting with the greatest barriers being unclear objectives about purpose and audience [24]. This lack of clarity creates debates about what information should be made available to consumers for decision-making and what is needed for clinical improvement with consumers seemingly not considered in designs or in some current reporting [24, 25]. Additionally, use of varying PREM instruments used across different facilities could also pose a problem for comparison by consumers and healthcare workers, alike.

Interestingly, some of the views expressed by hospital staff in this study around the perceived usefulness of PREMs as a decision-making tool for consumers were similarly expressed by healthcare consumers themselves. For instance, consumers often prefer referrals from friends/family or physicians over information obtained from the internet and they also acknowledged limitations on access based on insurance coverage and/or doctor privileges, thus possibly limiting their utility [26–28]. Concerns about the inability of hospitals to respond to negative reviews was also flagged elsewhere [26]. Consumer literacy was also an important consideration noted in this study if using PREMs as a consumer decision making tool for hospital care with previous studies also observing this as a limitation of current public hospital performance reports [24]. Future research should examine how consumers understand and interpret publicly available PREMs data and how this could be better communicated. Exploration of the views of front-line hospitals workers could also be explored in future research on their use and interpretation of PREMs data in their role, perhaps using a similar approach as to that used by Preng et al. (2018) with general practitioners.

A report on the falling growth in the use of private hospitals in Australia noted that no objective information on quality of care is available to private health consumers with the onus being on the consumer to find and interpret the information rather than providers routinely providing this data as a condition of receiving public funds [9]. Reporting of public hospital performance data has been mandated since 2011 in Australia. The lack of mandatory reporting by private hospitals has been noted by consumers in a previous study as “*ironic*” given that from a consumer perspective, public reporting of hospital performance would be most beneficial for the private system as this is where consumers have the most choice in hospital [24]. Participants in our study called for mandatory and standardised collection and reporting of PREMs across the private hospital system, with publication of PREMs being managed by Government Departments to improve trustworthiness.

### Strengths and limitations

Given the significance of organisational culture and leadership in patient experience initiatives and quality improvement, the views of healthcare organisation staff working in corporate or quality improvement roles in the Australian private healthcare sector is crucial.

Not all Australian private healthcare providers were approached or agreed to participate in this study, therefore this study may be representing the views of organisations who have a favourable PREMs culture and may not be applicable to other jurisdictions. Whilst all participants had a background as healthcare professionals (e.g., nurses), they were all currently working in corporate or patient experience roles. Views expressed in the current study may differ to staff whose roles are focused on administering clinical care.

## Conclusions

PREMs are viewed as an important tool for improving patient care and were regularly collected and reported for use by hospital staff for this purpose. Reporting of PREMs to consumers requires further research and development so that the nature of the collected data accurately reflects the needs of consumers and is presented in a fair and easily understood manner.

## Electronic supplementary material

Below is the link to the electronic supplementary material.

Supplementary Material 1

## Data Availability

The datasets used and/or analysed during the current study are available from the corresponding author on reasonable request.

## Footnotes

1 https://www.medibank.com.au/health-support/hospital-assist/experience-scores/.

2 https://www.aihw.gov.au/reports-data/myhospitals.
