# Supplementary material for: Australian private healthcare staff perspectives on patient reported experience measures (PREMs): a qualitative interview study
Source: J Patient Rep Outcomes. 2024 Nov 6;8:127. doi: 10.1186/s41687-024-00809-6 (PMC11541965; doi:10.1186/s41687-024-00809-6)
Supplement: Supplementary file 1 — Supplementary Material 1 [file 41687_2024_809_MOESM1_ESM.docx]

**Appendix A:** Hospital Staff Interview Schedule

**Understanding consumers’ and hospitals’ perspectives of Medibank patient reported experience measures (PREMs) data: a qualitative interview study**

**Hospital Staff Interview Schedule**

**Introduction**

Thank you very much for taking the time to do this interview.

The main purpose of this interview is to better understand how the private hospital you work at uses PREMS information and specifically whether / how the Medibank PREMS information is utilised. We are interested in your views on how to improve PREMS reporting for consumers and hospitals.

The interview is fairly informal in terms of structure, so if you have any questions or additional comments to add throughout the interview, please feel free to do so. It should take 30-60 minutes.

I have received your consent form. Thank you for completing that and sending it through.

Also, as mentioned in the Participant Information Sheet, this interview will be audio-recorded. This is to ensure that we have an accurate record of your responses, and everything you say will be kept strictly confidential. We will not report any identifiable information about you or the hospital you work at.

You are welcome to not state the name of your hospital throughout the interview.

Do you have any questions before we begin?

Okay, so I will start the audio-recording now.

**Hospital Staff Characteristics**

Just before we start talking about your perspectives on PREMS, I’ve got a few quick questions to help me know a characterise you and the hospital you work for generally. When reporting the results of this study we will always make sure your privacy and confidentiality is protected.

What is your professional background?

Qualifications:

Current Job title:

Years’ experience in healthcare:  ___________

Years experience as hospital executive / quality / patient experience manager:  __________

What State / Territory are you located in

□ NSW

□ VIC

□ QLD

□ WA

□ SA

□ TAS

□ NT

□ ACT

What is the population of the city/town you work in?

□ 1 million persons or more

□ 250,000 – 999,999 persons

□ 48,000 - 249,999 persons

□ 18,000 - 47,999 persons

□ 5,000 - 17,999 persons

□ 1,000 - 4,999 persons

□ less than 1000 persons

What is the size of the private hospital you work in

□ 400 or more beds

□ 300-399 beds

□ 200-299 beds

□ 100-199 beds

□ 50-99 beds

□ less than 50 beds

Does the private hospital you work in have an emergency department

□ Yes

□ No

Does the private hospital you work in have an ICU

□ Yes

□ No

Does the private hospital you work in provide maternity/obstetrics services

□ Yes

□ No

**Interview Questions**

**Access to PREMS data**

- What type of PREMS data is collected by your hospital?
- *Do you use the AHPEQS?* Australian Hospital Patient Experience Question Set (AHPEQS) is a tool with 12 questions that patients answer
- *Do you use the Australian Bureau of Statistics PEx (Patient Experience Survey)?*

- What type of PREMS data is provided to your hospital from other sources?
- *Do the PREMS measures vary? Do you use all of the different information?*
- *What other patient experience information do you receive?*

**Usage of PREMS**

- How are PREMs used in your health service?
- *What data do you most rely upon?*
- *How are the Medibank PREMS used?*
- *Do you review the Medibank PREMS data and compare to other PREMS sources?*
- *Do you utilise Medibank PREMS data to improve performance/address problem areas?*
- Does the reporting of PREMs result in any changes to processes, policies, staff behaviours?
- *If so, how?*

**Attitudes towards PREMS**

- How do you feel about PREMS data?
- *How do you feel about PREMS data as feedback / quality improvement*
- *How do you feel about PREMS data as a consumer health tool – providing information to help select hospitals*
- *What are your attitudes towards the Medibank PREMS data?*
- *How do you feel about the publication of this information on the internet? E.g. Medibank Hospital Experience Score website*
- What do you believe is your hospital and organisation’s attitudes towards PREMS?
- What are the advantages of receiving PREMS data?
- What are the disadvantages of receiving PREMS data?
- What information outside of PREMs would be of value to improve quality and patient experience?
- *e.g. publishing of out of pocket costs, PROMs, clinical outcomes, written patient feedback?*
- *What are the enablers and barriers to having these collected/published?*

**Medibank PREMS website**

- How do you feel about PREMS information about a hospital being made publicly available for health consumers?
- Are you aware of the Medibank Hospital Experience Scores website for healthcare consumers/patients?
- How do you feel about the current form of PREMS publishing on the Medibank website?
- *Can you tell me about the good and not so good elements of this website?*
- *Could this information be more meaningful? If so, how*
- *What changes would you suggest?*
- Are there any other PREMS websites that you believe are useful for health consumers?
- *Please discuss why these are helpful*

**Closing**
